# Inducible T-Cell Costimulator Ligand Plays a Dual Role in Melanoma Metastasis upon Binding to Osteopontin or Inducible T-Cell Costimulator

**DOI:** 10.3390/biomedicines10010051

**Published:** 2021-12-27

**Authors:** Davide Raineri, Giuseppe Cappellano, Beatrice Vilardo, Federica Maione, Nausicaa Clemente, Elena Canciani, Elena Boggio, Casimiro Luca Gigliotti, Chiara Monge, Chiara Dianzani, Renzo Boldorini, Umberto Dianzani, Annalisa Chiocchetti

**Affiliations:** 1 Dipartimento di Scienze della Salute, Interdisciplinary Research Center of Autoimmune Diseases-IRCAD, Università del Piemonte Orientale, 28100 Novara, Italy; davide.raineri@uniupo.it (D.R.); giuseppe.cappellano@med.uniupo.it (G.C.); beatrice.vilardo@uniupo.it (B.V.); federica.maione@med.uniupo.it (F.M.); nausicaa.clemente@med.uniupo.it (N.C.); elena.canciani@uniupo.it (E.C.); elena.boggio@med.uniupo.it (E.B.); luca.gigliotti@med.uniupo.it (C.L.G.); annalisa.chiocchetti@med.uniupo.it (A.C.); 2 Center for Translational Research on Autoimmune and Allergic Diseases, University of Piemonte Orientale, 28100 Novara, Italy; 3 Dipartimento di Scienza e Tecnologia del Farmaco, Università di Torino, 10125 Torino, Italy; chiara.monge@unito.it (C.M.); chiara.dianzani@unito.it (C.D.); 4 Divisione di Anatomia Patologica, Dipartimento di Scienze della Salute, AOU Maggiore della Carità, Università del Piemonte Orientale, 28100 Novara, Italy; renzo.boldorini@med.uniupo.it; 5 Laboratorio di Biochimica Clinica, Dipartimento di Scienze della Salute, AOU Maggiore della Carità, Università del Piemonte Orientale, Corso Mazzini 18, 28100 Novara, Italy

**Keywords:** osteopontin, ICOSL, melanoma, metastasis, tumor microenvironment

## Abstract

Recently, we demonstrated that inducible T-cell costimulator (ICOS) shares its unique ligand (ICOSL) with osteopontin (OPN), and OPN/ICOSL binding promotes tumor metastasis and angiogenesis in the 4T1 breast cancer model. Literature showed that OPN promotes melanoma metastasis by suppressing T-cell activation and recruiting myeloid suppressor cells (MDSC). On the opposite, ICOS/ICOSL interaction usually sustains an antitumor response. Here, we engineered murine B16F10 melanoma cells, by transfecting or silencing ICOSL. In vitro data showed that loss of ICOSL favors anchorage-independent growth and induces more metastases in vivo, compared to ICOSL expressing cells. To dissect individual roles of the three molecules, we compared data from C57BL/6 with those from OPN-KO, ICOS-KO, and ICOSL-KO mice, missing one partner at a time. We found that OPN produced by the tumor microenvironment (TME) favors the metastasis by interacting with stromal ICOSL. This activity is dominantly inhibited by ICOS expressed on TME by promoting Treg expansion. Importantly, we also show that OPN and ICOSL highly interact in human melanoma metastases compared to primary tumors. Interfering with this binding may be explored in immunotherapy either for nonresponding or patients resistant to conventional therapies.

## 1. Introduction

Melanoma is a malignant tumor arising from melanocytes that accounts for only about 1% of skin cancers [1] but it causes the large majority of skin cancer deaths [2]. Indeed, diagnosis is often made at late stages when melanoma cells have already metastasized. Melanoma is a highly immunogenic tumor, and infiltration with cytotoxic CD8^+^ T cells has been associated with a good prognosis, since they can efficiently kill tumor cells [3]. Melanoma immune escape is primarily due to alterations in antigen expression or presentation, but a role has been ascribed also to immunosuppression, mediated in particular regulatory T cells (Tregs) [4,5] and myeloid-derived suppressor cells (MDSCs) [6,7]. Both Tregs and MDSCs were found to be enriched and activated in the melanoma microenvironment, and are responsible for profound impairment of antitumor immune responses, leading to tumor progression [8].

Melanoma progression is associated with increased expression of osteopontin (OPN), a phosphoprotein acting either as a matricellular protein or as a proinflammatory cytokine [9]: biopsies from different stages of melanoma progression indicate that OPN is specifically overexpressed in invasive tumor stages [10]. Furthermore, high levels of OPN are significantly associated with poor clinical outcome in patients bearing highly metastatic tumors [11]: stromal OPN directly suppresses CD8^+^ T cell proliferation and activation [12], and in parallel, sustains MDSCs colonization, thus supporting tumor progression [13].

The role of OPN in melanoma metastasis has been demonstrated clearly in animal models. B16-F10 is a murine melanoma cell line used as a model to study metastasis [14]. Injection of B16-F10 melanoma cells in the tail vein of syngeneic mice results in extravasation into the lungs and formation of countable macro-metastases [14]. The intracardiac injection of B16-F10 cells leads to metastasis in the lungs and other organs, such as the liver [15]. Intracardiac injection of B16-F10 cells into OPN knock-out (OPN-KO) mice produces fewer metastases than occur in wild-type mice, whereas no difference is found when injection is intravenous [16]. 

OPN is expressed by different components of the tumor microenvironment, such as macrophages, dendritic cells (DCs), and activated T cells. Thanks to its multiple adhesion motifs (i.e., calcium-binding sites, heparin-binding domains and integrin domains), OPN is able to interact with different receptors and exerts multiple functions. In particular, two classes of receptors have long been identified for OPN, namely integrins and CD44_v6-7_ [17]. More recently, for the first time, the present group demonstrated that OPN directly binds the immune checkpoint receptor inducible T-cell costimulator ligand (ICOSL) [17], finding that it binds directly to ICOSL at a binding site that is distinct and nonoverlapping compared to that employed by ICOS, which is the conventional partner of ICOSL. The two interactions exert opposing effects on cell migration, which is induced by OPN, but dominantly inhibited by ICOS. Moreover, angiogenesis and tumor metastasis are increased, both in vitro and in vivo, by OPN-mediated triggering of ICOSL, whereas they are inhibited by ICOS-mediated ICOSL triggering [17,18,19].

ICOSL is a transmembrane receptor belonging to the B7 family that is expressed in B cells, dendritic cells, monocytes, endothelial cells, fibroblasts, epithelial cells, and several types of tumor cells [20]. Its main known function is to trigger ICOS, a costimulatory receptor expressed by activated T cells [21]. Inflammatory signals increase ICOSL expression in several cell types of nonlymphoid tissue, such as the brain, lung, heart, kidney, liver, and gut, which suggests that the ICOS/ICOSL interaction regulates the activity of effector and effector/memory T cells [22]. ICOS is a type I transmembrane glycoprotein belonging to the CD28 family; it acts as a costimulatory immune checkpoint receptor. It is expressed by activated T cells, and its stimulation modulates differentiation and potentiates cytokine production of T helper (Th) cells [21]. Depending on the microenvironment cytokine milieu, ICOS supports differentiation of Tregs: Th17, Th2 (in mouse), and Th1 (in human) cells. Moreover, it is highly expressed by T follicular helper (Tfh), and a deficiency of the ICOS gene causes defective formation of germinal centers in both humans [23]. The ICOS-ICOSL interaction also triggers “reverse signaling” to the cell expressing ICOSL. In DCs, triggering of ICOSL with ICOS-Fc, a recombinant soluble form of ICOS, modulates cytokine secretion, promotes antigen cross-presentation, and inhibits adhesiveness and migration; in endothelial and tumor cells it inhibits adhesion and migration. Moreover, it induces dephosphorylation of ERK and p38 in endothelial cells, dephosphorylation of FAK in tumor cells, and downmodulation of β-PIX in DCs and tumor cells [19,24,25,26]. The present group employed the B16 mouse melanoma model to demonstrate that ICOS-Fc inhibits cell migration in vitro and the development of metastases in vivo [19,24]. Further, ICOS-Fc encapsulated in nanoparticles inhibits the growth of established subcutaneous B16 tumors by inhibiting tumor angiogenesis and Treg development [18].

Collectively, these data draw a picture in which the triggering of ICOSL by one or other of the binding partners, ICOS and OPN, mediates opposing activities on tumor metastasis, which is promoted by OPN and inhibited by ICOS. Stemming from these findings, the present study aimed to evaluate the net effect of ICOSL binding to OPN and to ICOS on the metastasis potential of the two binding partners in vivo. To this end, B16-F10 melanoma cells, stably silenced for ICOSL, were injected intravenously (i.v.) into either syngeneic wild-type C57BL/6 mice, or mice deficient in one molecule at a time (OPN-KO, ICOS-KO, ICOSL-KO mice). Interestingly, it emerged that OPN, through binding with ICOSL, promotes melanoma metastasis formation.

## 2. Materials and Methods

### 2.1. Cell Lines

B16-F10 mouse melanoma cells, purchased from the American Type Culture Collection (ATCC; Manassas, VA, USA), were grown in culture dishes as a monolayer in RPMI 1640 medium plus 10% fetal bovine serum (FBS) (Life technologies, Carlsbad, CA, USA), 100 U/mL penicillin, and 100 μg/mL streptomycin at 37 °C in 5% CO_2_-humidified atmosphere. 

### 2.2. ICOSL Cloning 

ICOSL was cloned as described elsewhere [17]. Briefly, cDNA derived from RNA extracted from mouse splenocytes was cloned into pcDNA 3.1 vector. The plasmid was then transformed into JM109 bacteria (Promega, Madison, WI, USA) and the resulting colonies were sequenced by Sanger sequencing (Life technologies, Carlsbad, CA, USA).

### 2.3. Cell Transfection and Cell Silencing

B16-F10 cells were transfected as reported elsewhere [17]. Briefly, 2 × 10^6^ B16-F10 cells were transfected with 10 μg of a DNA plasmid (pcDNA3.1) carrying the ICOSL cDNA, using lipofectamine^TM^ 3000 (Life technologies, Carlsbad, CA, USA). The endogenous ICOSL gene was siRNA-silenced using lipofectamine^TM^ RNAiMAX transfection reagent (Life technologies, Carlsbad, CA, USA) as reported elsewhere [17]. Real-time PCR was used to evaluate the expression of ICOSL in the transfected (B16-ICOSL-high) and silenced (B16-ICOSL-low) cells, 48 h after transfection and silencing. Next, 1 μg of RNA was retrotranscribed to cDNA using the QauntiTect Reverse Transcription Kit (Qiagen, Hilden, Germany). Real-time PCR was performed using CFX96 System (Bio-Rad Laboratories) in duplicate for each sample, in a 10 μL final volume containing 1 μL of diluted cDNA, 5 μL of TaqMan Universal PCR Master Mix (Life technologies, Carlsbad, CA, USA), and 0.5 μL of Assay-on-Demand mix. The results were analyzed with a ΔΔ threshold cycle method using *GAPDH* as housekeeping gene. Surface expression of ICOSL was evaluated by flow cytometry using anti-ICOSL PE mAb (Catalog number #107405, clone HK5.3, Biolegend, San Diego, CA, USA).

### 2.4. Invasion Assays 

In the Boyden chamber (BD Biosciences) invasion assay, 1 × 10^3^ cells of B16-ICOSL-high and B16-ICOSL-low were plated onto the apical side of 50 µg/mL Matrigel-coated filters (8.2 mm diameter and 0.5 mm pore size; Neuro Probe; BIOMAP snc, Milan, Italy) in serum-free medium with or without 2 µg/mL ICOS-Fc. Medium containing 20% FBS, as control, or 10 μg/mL of OPN (Bio-techne, Minneapolis, MN, USA) were placed in the basolateral chamber as chemoattractants for the tumor cells. The chamber was incubated at 37 °C under 5% CO_2_. After 6 h, the cells on the apical side were wiped off with Q-tips. The cells on the bottom of the filter were stained with crystal violet and all counted with an inverted microscope (magnification ×40). The results are expressed as the number of migrated cells per high-power field. 

### 2.5. Anchorage-Dependent Growth Assay

For the anchorage-independent growth assay, 1.6 × 10^3^ cells were plated in RPMI (Life technologies, Carlsbad, CA, USA) plus 10% FBS in a 96-well plate. From day 1 to day 4 cells were counted daily using trypan blue. For the anchorage-independent growth assay, a 12-well plate was coated with RPMI 3% agar. After solidification, 5 × 10^3^ cells were plated over the 3% agar bottom layer, in RPMI 1% agar. Cells were grown at 37 °C for 14 days. 1 mg/mL of 3-(4,5-dimethylthiazol-2-yl)-2,5-diphenyltetrazolium bromide (MTT) was then added and the plate incubated for 3 h at 37 °C. The colony images were taken using Chemidoc (Biorad, Hercules, CA, USA).

### 2.6. In Vivo Experiments

Eight-to-ten-week-old adult female mice of the following 4 standard inbred strains were used: C57BL/6, B6.129S6(Cg)-Spp1tm1Blh/J, also known as OPN KO, B6.129P2-Icostm1Mak/J, also known as ICOS KO, and B6.129P2-Icosltm1Mak/J, also known as ICOSL KO (all mice were purchased from The Jackson Laboratory, Bar Harbor, ME, USA). Animals were maintained under pathogen-free conditions in the animal facility of Università del Piemonte Orientale; they were fed ad libitum on rodent chow, and water was freely available in the home cages; the ambient temperature was maintained at 21 ± 1 °C. All experimental procedures were conducted during the light phase of a 12:12 h light:dark cycle. One million cells were i.v. injected and after 15 days the mice were sacrificed and lung metastases counted by two operators working blind. The lungs were cut into small pieces and incubated at 37 °C for 30 min with 0.5 mg/mL of Collagenase type IV (Merck, Darmstadt, Germany). After neutralization of the enzyme with 2 mM of EDTA (Merck, Darmstadt, Germany), the tissue was crushed and passed through a 100 μm filter. Red blood cells were lysed by osmotic shock. Aliquots of one million cells were stained with mAb as follows. Myeloid MDSC (M-MDSC) (CD11b^+^, Ly6C^high^ and Ly6G^low^) with anti-CD11b Percp-Cy5.5 (Catalog number #101228, clone M1/70, Biolegend, San Diego, CA, USA), anti-Ly6C APC (Catalog number #128016, clone HK1.4, Biolegend, San Diego, CA, USA), and anti-Ly6G APC-Cy7 (Catalog number #127624, clone 1A8, Biolegend, San Diego, CA, USA) Treg cells (CD4^+^, CD25^+^ and Foxp3^+^) with anti-CD4 FITC (Catalog number #100406, clone GK1.5, Biolegend, San Diego, CA, USA) and anti-CD25 PE (Catalog number #12-0251-83, clone PC61.5, Life Technologies, Carlsbad, CA, USA); cells were then permeabilized and stained with anti-Foxp3 APC antibody (Catalog number #17-5773-82, clone FJK-16s, Life Technologies, Carlsbad, CA, USA). Cells were acquired using Attune flow cytometry (Thermo Fisher Scientific, Waltham, MA, USA) and data analyzed using FlowJo Software (Becton and Dickinson, Franklin Lakes, NJ, USA). The results are expressed as % of Ly6C^high^ in CD11b^+^ cells (M-MDSC) and as % of Treg in CD4^+^ cells (Treg).

### 2.7. Proximity Ligation Assays (PLA)

PLA technology is based on the detection of protein interactions. This technique uses one pair of primary antibodies that target the proteins of interest with the aim of studying the interaction. PLA was carried out with Duolink^®^ In Situ Red Starter Kit Goat/Rabbit (Merck, Darmstadt, Germany). Additional reagents used were Duolink^®^ In Situ PLA^®^ Probe Anti-Goat MINUS (# DUO92006) and Duolink^®^ In Situ PLA^®^ Probe Anti-Rabbit PLUS (#DUO92002). Antibody concentrations were optimized using immunofluorescence prior to PLA experiments. Human melanocytic skin lesions or metastatic tissue have been removed based on the clinical suspicion/confirmation of melanoma, accordingly to the best clinical practices (study approved by the Ethical Committee of AOU Maggiore della Carità di Novara, 14 December 2012, CE 166/12). The origin of the tumor biopsy (i.e., primary tumor or metastasis) was confirmed by the pathologist. Each primary tumor was paired with its lymph node metastasis. No clinical data are provided in the manuscript because samples were anonymized. Paraffin-embedded primary tumor or metastasis tissues from patients were deparaffinized and subjected to graded rehydration through an alcohol series (xylene; 100, 95, and 70% ethanol) before immersion in PBS 1×. Heat-induced epitope retrieval was performed using a microwave. Tissue samples were then blocked with protein block serum-free (Dako) for 1 h at room temperature. Tissues were incubated with primary antibodies (diluted 1:100 in blocking solution) overnight at 4 °C. The In Situ Red Starter Kit Goat/Rabbit was used following the manufacturer’s protocol. The sections were observed by Leica TCS SP2 AOBS confocal laser-scanning microscope (Leica Microsystems, Wetzlar, Germany) and analyzed with the Image Pro Plus Software for micro-imaging 5.0 (Meyer Instruments, Houston, TX, USA).

### 2.8. Statistics

The number of metastases, the infiltrate among the mouse groups, and migrations were analyzed using one-way ANOVA; in all other cases the Mann–Whitney *t*-test was used as indicated. *p* values below 0.05 were considered statistically significant. The statistical analyses were performed with GraphPad Instat software (Prism 8 version 8.4.3) (GraphPad Software, San Diego, CA, USA). 

## 3. Results

### 3.1. OPN and ICOSL Highly Interact in Human Melanoma Metastases

**Figure 1:**
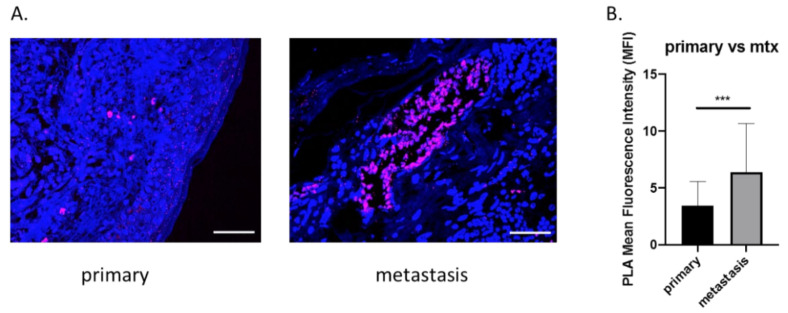
Increased OPN-ICOSL interaction in primary and its paired metastatic human melanoma. (**A**) Proximity Ligation Assay (PLA) in primary tumor and corresponding metastasis. The merged PLA signal (violet) shows ICOSL and OPN interaction, while nuclei are labeled with DAPI (blue). Images were quantified using ImageJ software in order to calculate the ratio between red (OPN) and green (ICOSL) channels; values are shown as percentage of red–green co-staining. Scale bar 50 μm. Four images were analyzed for each sample at a magnification of 40×. (**B**) The histograms quantify OPN-ICOSL PLA in signals of primary and metastatic tumors, expressed as MFI (mean ± SEM). Mann–Whitney statistical test was used, *** *p* < 0.001.

Recently, in a murine breast cancer model, we showed that OPN interacts with ICOSL, and that this binding sustains the angiogenic process. To characterize the role of OPN-ICOSL in patient-derived tumors more precisely, human melanoma samples were here scored for mutual OPN/ICOSL interactions. To this end, the proximity ligation assay (PLA) technology was employed, as it is a highly specific and sensitive tool for the in situ detection of protein-protein interactions [17]. By means of immunofluorescence (IF) analysis, human specimens of primary melanomas (*n* = 10) and their matched lymph node metastases (*n* = 10) were screened; OPN/ICOSL interactions were found in both primary tumor and metastatic tissues. Quantification of the OPN-ICOSL PLA signal showed this was higher in metastases than in primary tumors (Figure 1A,B). 

These findings suggest that the simultaneous presence of OPN and ICOSL is relevant in human melanomas, and that binding between these two molecules increases in metastatic lesions.

### 3.2. ICOSL Expression Modulates In Vitro Growth and In Vivo Metastasis of B16-F10 Melanoma Cells

Since OPN/ICOSL were observed to increase in human metastatic melanomas, it was decided to investigate whether the interaction between these two molecules could also play a role in a preclinical B16 mouse model. To this end, B16-F10 cells were injected i.v. into syngeneic mice to generate black countable lung colonies.

**Figure 2:**
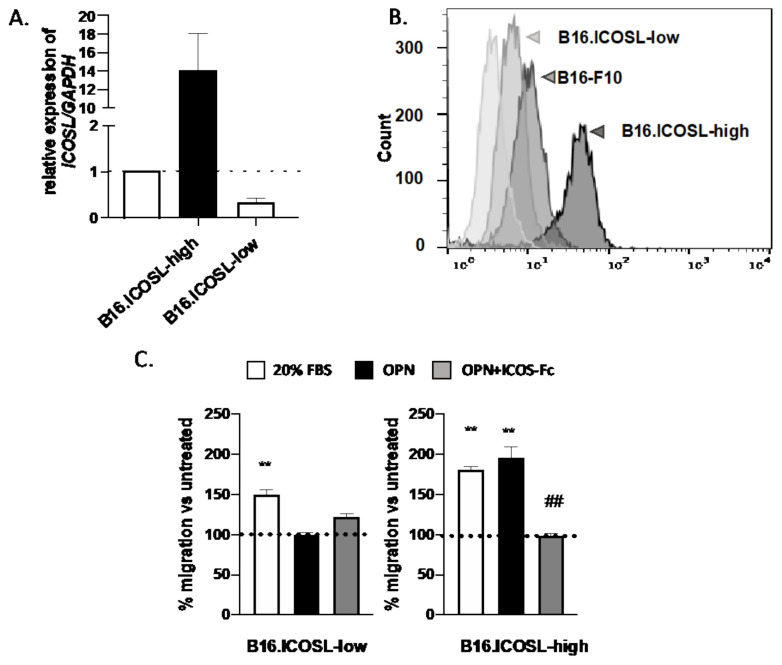
Engineering of B16-F10 melanoma cell lines expressing stable levels of ICOSL. (**A**) Relative quantification of *ICOSL* RNA in B16-ICOSL-high and B16-ICOSL-low cells; the dotted line shows B16-F10 parental cells. Data are shown as means ± SEM gene expression relative to expression of the endogenous control *GAPDH* (2^−ΔCt^ method). One representative experiment is shown (*n* = 3 biological replicates); (**B**) representative histogram showing surface expression of ICOSL in B16-ICOSL-high (dark gray), B16-ICOSL-low (gray), and parental B16-F10 cells (light gray); (**C**) migration of B16-ICOSL-high and B16-ICOSL-low cells in response to 10 μg/mL of rhOPN or 20% FBS as chemotactic stimulus. The gray column shows inhibition of OPN-induced migration by ICOS-Fc. The dotted line shows the migration of control cells, set at 100% in the absence of chemotactic stimuli. Data are expressed as means ± SEM (*n* = 5) of the number of migrated cells per high-power field, ** *p* < 0.01 refers to baseline, ## *p* < 0.01 refers to OPN-induced migration, one-way ANOVA test was used.

Of note, B16-F10 parental cells express ICOSL, as shown by RT-PCR (Figure 2A) and flow cytometry (Figure 2B), but do not express ICOS or OPN (data not shown). Since surface expression of ICOSL was variable in different cell batches, and was closely dependent on cell culturing conditions (not shown), cells were engineered to stably express high (B16-ICOSL-high) or low (B16-ICOSL-low) levels of ICOSL. B16-ICOSL-high cells were obtained by transfecting B16-F10 cells with a plasmid encoding the entire murine ICOSL cDNA, and B16-ICOSL-low by transfecting B16-F10 cells with a siRNA targeting ICOSL. Both cell lines showed stable expression levels of ICOSL mRNA and protein, which were consistently higher in B16-ICOSL-high than in B16-ICOSL-low cells (Figure 2A,B). A previous study having found that melanoma cell migration in response to OPN depends on ICOSL expression, the migration response to OPN of the two cell lines was compared (Figure 2C). B16-ICOSL-high cells were seen to migrate toward OPN, whereas B16-ICOSL-low cells did not. As expected, ICOS-Fc was able to interfere and block this migration (Figure 2C).

**Figure 3:**
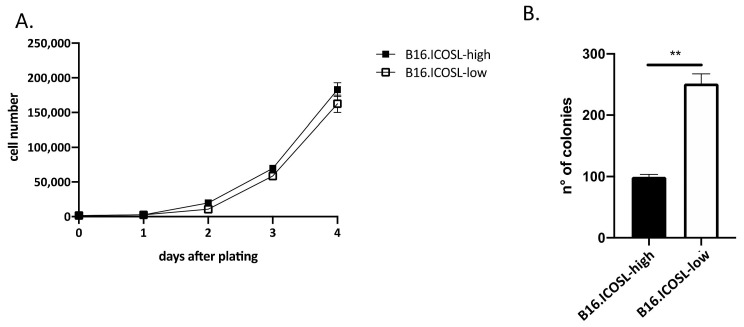
ICOSL expression inhibits anchorage-independent tumor cell growth. (**A**) Kinetic growth assay of B16-ICOSL-low and B16-ICOSL-high cells in the presence of 10% FBS (*n* = 2). Cells were counted following trypan blue staining; (**B**) the soft agar colony formation assay was run to estimate anchorage-independent growth of B16-ICOSL-low and B16-ICOSL-high cells. Colonies were counted digitally using ImageJ software. Data are expressed as means ± SEM. The Mann–Whitney statistical test was used, ** *p* < 0.01.

In previous work, it was found that ICOSL expression inhibits the growth of mammary carcinoma cells in anchorage-independent but not in anchorage-dependent conditions, thus the in vitro growth of B16-ICOSL-high and of B16-ICOSL-low cells in these culture conditions was compared. B16-ICOSL-high and B16-ICOSL-low displayed comparable anchorage-dependent growth, as assessed by culturing cells in standard culture plates in the presence of FBS (Figure 3A). However, anchorage-independent growth in soft agar was lower in B16-ICOSL-high cells than in B16-ICOSL-low cells (Figure 3B). 

**Figure 4:**
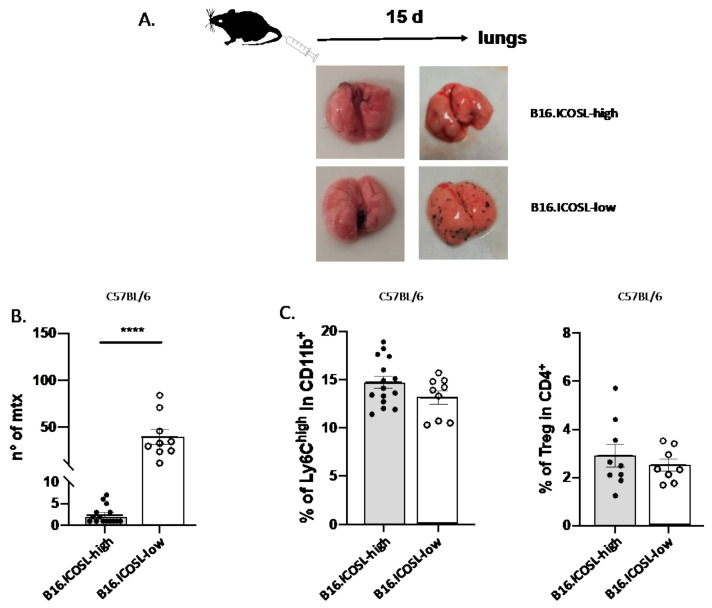
Role of ICOSL expression in B16-F10 cell metastasis. (**A**) Schematic illustration of experimental in vivo approach (top) and representative images of metastatic lung (bottom) from B16-ICOSL-high and B16-ICOSL-low injected mice, at baseline (left) and after 15 days (right). (**B**) The graph shows the number of lung metastases in B16-ICOSL-high and B16-ICOSL-low C57BL/6-injected mice (*n* = 9–15 mice). Data are shown as means ± SEM. The Mann–Whitney statistical test was used, **** *p* < 0.0001 (**C**) Left: bar graph showing the percentage of M-MDSC among total CD11b^+^ cells in the lung of C57BL/6 mice injected with B16-ICOSL-high and B16-ICOSL-low cells. Right: bar graph showing the percentage of Treg among total CD4^+^ cells in the lung of C57BL/6 mice injected with B16-ICOSL-high and B16-ICOSL-low cells.

The finding that tumor cells expressing low levels of ICOSL grow efficiently in the absence of anchorage to the extracellular matrix suggests that they may be prone to metastasize in vivo. To assess this possibility, the metastatic potentials of B16-ICOSL-high and B16-ICOSL-low cells were compared, injecting them into the tail vein of C57BL/6 mice and counting lung metastases after 15 days: B16-ICOSL-low cells produced more metastases than B16-ICOSL-high cells (Figure 4A,B). In order to exclude any nonlung metastatic spread, we macroscopically analyzed all the available organs comprising liver, omentum and gut, lymph nodes, muscles, bone and brain: no metastases were found (data not shown). The TME of the metastases was characterized by analyzing the infiltrating leukocytes of the metastatized lung, focusing on M-MDSC and Tregs, which are known to be supported by OPN and ICOS, respectively, and are key components of the immune suppressive TME [13,27,28,29]. M-MDSC were evaluated as percentage of CD11b^+^/Ly6C^+^/Ly6G^−^ cells among total CD11b^+^ cells, and Treg cells as percentage of CD4^+^CD25^+^Foxp3^+^ among total CD4^+^ cells (the gating strategy and the fluorescence minus controls (FMOs) are shown in Supplementary Figures S1 and S2). Similar amounts of both cell types were detected in lungs metastasized with either B16-ICOSL-low or B16-ICOSL-high cells (Figure 4C), suggesting that the metastasis process, in this setting, is not influenced by recruitment of M-MDSC and Tregs in premetastatic niches and finally, the percentages are in line with those detected in the lungs of tumor-free mice (Supplementary Figure S3).

### 3.3. ICOSL Mediates Different Metastatic Effects When Binding to OPN or to ICOS

With C57BL/6 mice it is not possible to model the net OPN/ICOSL interaction in the tumor context, since these mice fully express all three molecules (OPN, ICOS, and ICOSL) and thus the effect of each may be masked by an opposing action of the others. Thus, in order to gain in-depth understanding of the roles of ICOSL’s two binding partners, and to elucidate their mechanisms, the metastasis of B16-ICOSL-high and B16-ICOSL-low cells was examined in wild-type mice compared to OPN-, ICOS-, or ICOSL-KO mice; these are mice in which either OPN or ICOS or ICOSL is lacking. ICOSL-KO mice were included to examine the role of stromal ICOSL in comparison to tumor ICOSL. When necessary, ICOSL expression was silenced in tumor cells to eliminate its interference, using B16-ICOSL-low cells. For comparison purposes, ICOSL was also transfected into B16-F10 cells, so as to overexpress it and evaluate its role when expressed by tumor cells, using B16-ICOSL-high cells. Of note, these engineered cell models are only capable of addressing OPN produced by stromal cells (B16-F10 cells do not secrete OPN; data not shown) whereas they can address ICOSL expressed either by tumor cells or by the stromal compartment. 

**Figure 5:**
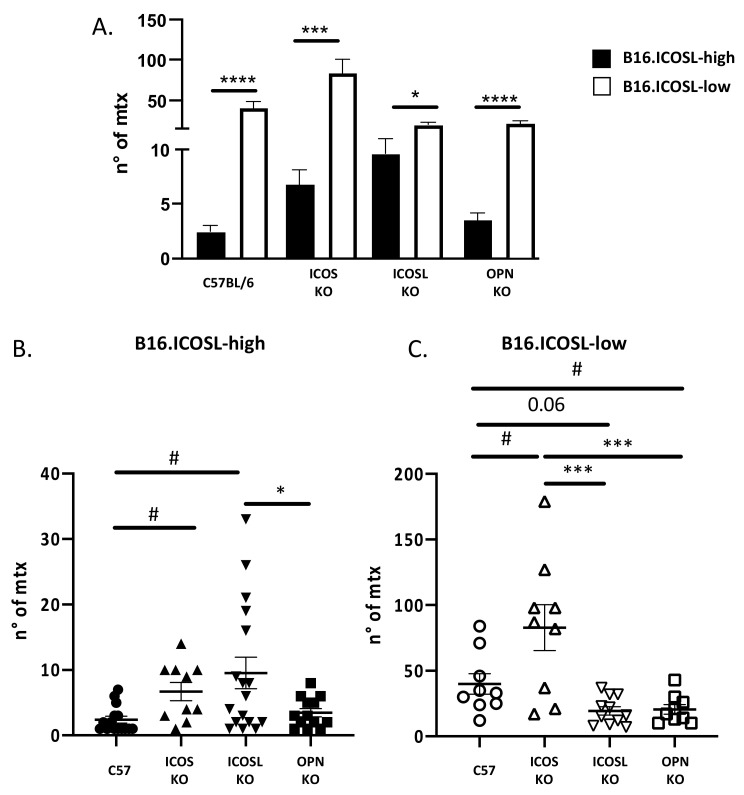
ICOSL mediates different metastasizing effects when binding to OPN and ICOS. (**A**) The histogram shows the number of metastases in each genotype (C57BL/6, OPN-KO, ICOSL-KO and ICOS-KO) of B16-ICOSL-high and B16-ICOSL-low injected mice (*n* = 9–17 mice). (**B**) The histogram shows the number of metastases in different genotypes (C57BL/6, OPN-KO, ICOSL and ICOS-KO) in B16-ICOSL-high injected mice (*n* = 9–17 mice). (**C**) The histogram shows the number of metastases within genotypes (C57BL/6, OPN-KO and ICOS-KO) of B16-ICOSL-low injected mice (*n* = 9–11 mice). One-way ANOVA test was used, * *p* < 0.05, *** *p* < 0.001, **** *p* < 0.0001 and # *p* < 0.05 refers to C57 mouse background.

The first comparison between B16-ICOSL-low and B16-ICOSL-high cells showed that the former produced more metastases than did the latter, in OPN-KO, ICOS-KO, and ICOSL-KO strains, and in wild-type mice (Figure 5A). 

The second analysis compared the metastases formed by B16-ICOSL-high, displaying low metastatic behavior, in the different mouse strains. Results showed that metastases were higher in ICOSL-KO mice and ICOS-KO mice than in wild-type mice. In addition, ICOSL-KO mice displayed an increased amount of metastasis compared to OPN-KO mice (Figure 5B). The results of ICOS-KO mice confirm that ICOS inhibits metastasis. By contrast, the results of ICOSL-KO and OPN-KO were unexpected and indicated that expression of high levels of ICOSL in the tumor cells increases the system complexity. 

The third analysis compared the metastases formed by B16-ICOSL-low, displaying high metastatic behavior, in the different mouse strains. Results showed that, compared to wild-type mice, metastases were decreased in OPN-KO mice and ICOSL-KO mice, and increased in ICOS-KO mice (Figure 5C). These results are in line with the model that OPN and ICOSL promote, whereas ICOS inhibits metastasis.

To assess the effects of ICOS, OPN and ICOSL expressed by the TME on the B16-F10 metastasis microenvironment, the lung-infiltrating m-MDSC and Treg cells in wild-type mice were compared with those in the different KO mice (the gating strategy is shown in Supplementary Figures S1 and S2).

**Figure 6:**
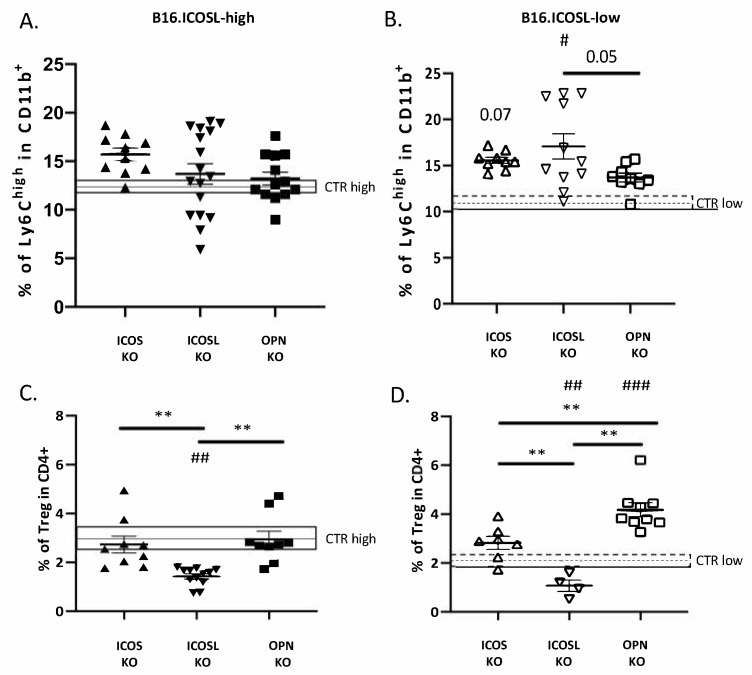
Percentage of Treg and M-MDSC infiltrating B16.ICOS-high/low tumors in C57BL/6 and KO mice (**A**) M-MDSC as % of CD11b^+^ cells in lungs of C57BL/6, OPN-KO, ICOSL-KO and ICOS-KO mice injected with B16-ICOSL-high cells; the gray rectangle shows the percentage of M-MDSC in C57 mice. Data are means ± SEM (*n* = 10–17 mice). (**B**) M-MDSC as % of CD11b^+^ cells in lungs of C57BL/6, OPN-KO, ICOSL-KO and ICOS-KO mice injected with B16-ICOSL-low cells; the gray rectangle shows the percentage of M-MDSC in C57 mice. Data are means ± SEM (*n* = 9–11 mice). (**C**) Treg as percentage of CD4^+^ in lungs of C57BL/6, OPN-KO and ICOS-KO mice injected with B16-ICOSL-high cells; the gray rectangle shows the percentage of Tregs as percentage of CD4^+^ in C57BL/6 mice. Data are means ± SEM (*n* = 9–12 mice). (**D**) Treg as percentage of CD4^+^ in lungs of C57BL/6, OPN-KO and ICOS-KO mice injected with B16-ICOSL-low cells; the gray rectangle shows the percentage of Tregs in C57BL/6 mice. Data are means ± SEM (*n* = 4–9 mice). One-way ANOVA test was used, ** *p* < 0.01; # *p* < 0.05, ## *p* < 0.01 and ### *p* < 0.001 refer to C57BL/6 mouse background.

As in wild-type C57BL/6 mice also in ICOS-KO, OPN-KO and ICOSL-KO mice, m-MDSC and Treg were not significantly different in lungs metastasized by B16-ICOSL-high versus B16-ICOSL-low cells (Figure 6). On the contrary, comparison of wild-type mice with each KO strain showed that: (1) M-MDSC were significantly increased in ICOS-KO and a trend (*p* = 0.07) was found in ICOSL-KO mice when injected with B16-ICOSL-low, but not with B16-ICOSL-high cells (Figure 6A,B); (2) Tregs were decreased in ICOSL-KO mice injected with both B16-ICOSL-high -low cells, and increased in OPN-KO mice with B16-ICOSL-low cells, but not in those injected with B16-ICOSL-high cells (Figure 6C,D). All the other comparisons showed no statistically relevant difference. 

The amounts of M-MDSCs and Tregs obtained from the lungs of control tumor-free mice were similar in wild type and each KO strain (Supplementary Figure S3).

## 4. Discussion

This study investigates the role in tumor cell metastasis of the ICOS/ICOSL/OPN network, comparing the metastasizing capability of two variants of melanoma B16-F10 cells, respectively expressing high and low levels of ICOSL. The study used wild-type mice and three strains of KO mice, lacking either ICOS, or OPN, or ICOSL. It emerged that the three members of the ICOS/ICOSL/OPN network play distinct and differing roles, possibly acting both at the tumor cell level and at that of the TME. These differing effects may be ascribed to the complexity of the ICOS/ICOSL/OPN network, in which (1) both ICOS and OPN bind to ICOSL, but exert different, often opposing, effects on the cell that expresses ICOSL; (2) OPN also binds other surface receptors, such as integrins and CD44; (3) OPN and its ligands (ICOSL, integrins, and CD44) can be expressed by tumor cells and by several cell types of the TME, influencing tumor cell survival, proliferation, and metastasis; (4) ICOS can be expressed by different types of T cells, including effector and Treg cells, and also by some other cell types in the TME, such as DCs and macrophages. Moreover, OPN produced by tumor cells may lack important domains and may carry different post-translational modifications, making it structurally and functionally different from that produced by other cell types [30].

The first part of the study validates the model employing two types of B16-F10 cells, and confirms the results of previous work on different tumor cell types [17]. That prior study showed that expression of high levels of ICOSL promotes cell migration in response to OPN, which is inhibited by ICOS-mediated triggering of ICOSL, and that it inhibits tumor cell growth in anchorage-independent, but not in anchorage-dependent, conditions. The effect on anchorage-independent growth suggests that expression of ICOSL may increase tumor cell anoikis, which is a cell-death process whereby cells undergo apoptosis after they lose contact with the extracellular matrix (ECM) [31]. Acquisition of anoikis resistance is necessary for tumor cells to survive while traveling through the circulation [32]. 

Since the triggering of ICOSL by OPN, or alternatively by ICOS, exerts opposing effects on tumor cell migration, metastasis of B16-ICOSL-high and of B16-ICOSL-low cells in wild-type mice was compared, to assess the net effect of these opposing forces in vivo. Expression of high levels of ICOSL was found to substantially decrease metastasis, suggesting that the inhibitory effect of ICOS-mediated triggering of ICOSL is dominant over the promoting effect mediated by OPN; this is in line with previous work showing that treatment of mice with ICOS-Fc inhibits tumor cell metastasis [18].

**Figure 7:**
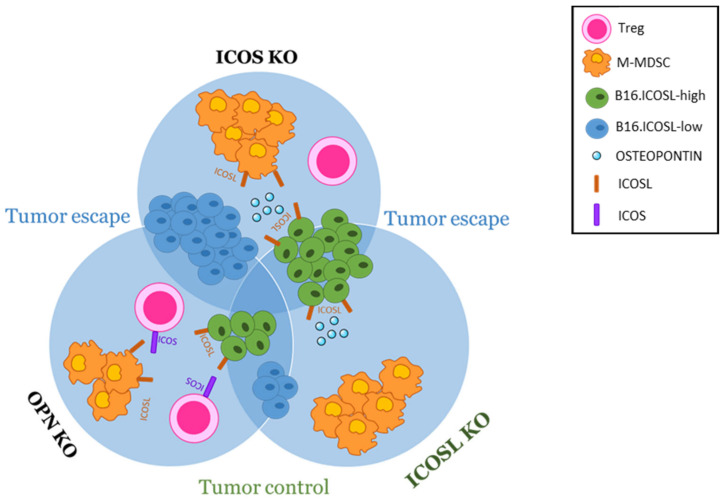
The metastatic niche model at a glance. The melanoma metastatic niche contains malignant cells towards which immune suppressive cells are recruited and corrupted. Tumor and stromal cells produce the ECM proteins that regulate cell function, and their interaction is also relevant for tumor progression and metastasis. The model used here was implemented to investigate metastasis, Tregs and M-MDSC-infiltrating metastatic melanoma, induced by ICOSL binding to ICOS or to OPN. Melanoma cells, expressing ICOSL (green) or not expressing it (blue), were used to induce metastases in scenarios lacking one of the three molecules of the trio. By removing one molecule at a time, the effect of each molecule expressed can be observed without interference from the other two. The results on the three strains of KO mice were then compared to each another and to those on wt mice. Using this model, it was shown that OPN/ICOSL promote metastasis.

To dissect the role of each member of the ICOS/OPN/ICOSL network expressed in the TME, the metastasis ability of B16-ICOSL-high was compared with that of B16-ICOSL-low cells, using KO mice lacking one network member at a time. B16-ICOSL-high and B16-ICOSL-low cells express neither OPN nor ICOS, so that the only source of the two ligands of ICOSL was in the TME. Importantly, removing each molecule of the trio, one at a time, is useful at a twofold level: on one hand it allows evaluation of the effect of the absence of the knocked-out molecule itself, but on the other hand it sets the conditions to evaluate, without any interference, the residual activity of the other two interactors, as depicted for better clarity in Figure 7. Analysis of ICOS-KO mice showed that, compared to wild-type mice, they undergo increased metastasis formation, whether using B16-ICOSL-high or B16-ICOSL-low cells. This effect is in line with the loss of the dominant ICOS-mediated triggering of ICOSL both on tumor or stromal cells. Indeed, in B16-ICOSL-low metastases, the increased numbers of M-MDSC cells might play a role in the increased metastasis, and help tumor cells (not expressing ICOSL) to elude the immune system. On the contrary, M-MDSC were not increased in B16-ICOSL-high metastases, suggesting that expression of high levels of ICOSL on tumor cells might inhibit the development of M-MDSC, possibly by sequestering OPN, known to support M-MDSC development. In line with this hypothesis, the same increase in M-MDSC displayed by B16-ICOSL-low, (and not by B16-ICOSL-high metastases), was also detected in ICOSL-KO mice, (and not in OPN-KO mice). Since C57BL/6 mice did not show any increase in M-MDSC, these data collectively suggest that such an increase requires both the availability of OPN and absence of the ICOS/ICOSL interaction at the TME level. 

Analysis of OPN-KO mice showed that, when compared to wild-type mice, they developed fewer metastases using B16-ICOSL-low. This effect is in line with a loss of the prometastatic effect exerted by OPN. Interestingly, this model shows that B16-ICOSL-low metastases contained increased numbers of Treg cells compared to C57BL/6 mice, suggesting that OPN may inhibit development of these cells; this effect would be reversed by the expression of high levels of ICOSL in B16-ICOSL-high cells, which is in line with the notion that ICOS triggering in T cells supports Tregs.

Lastly, through analysis of ICOSL-KO, the role played by ICOSL—expressed by either the tumor or the TME cells—was elucidated. Compared to C57BL/6, ICOSL-KO mice displayed more B16-ICOSL-high metastases and fewer B16-ICOSL-low metastases, which runs counter to the dominant negative effect exerted by ICOS on adhesion and migration of ICOSL-expressing tumor cells. One possible explanation for this disparity is that, in ICOSL-KO mice, the lack of endogenous ICOSL makes large amounts of OPN available to the tumor cells, an abundance that might promote metastasis of B16-ICOSL-high cells by overcoming the dominant negative effect exerted by ICOS. Importantly, low numbers of B16-ICOSL-low metastases, which were comparable in number to those occurring in OPN-KO mice, suggest that the metastasis process is closely related to the simultaneous expression and interaction of OPN and ICOSL. In ICOS-KO mice, OPN has no competitor for ICOSL in the TME in promoting M-MDSC recruitment. Moreover, the observation that both B16-ICOSL-high and B16-ICOSL-low metastases displayed lower numbers of Treg cells in ICOSL-KO mice than they did in wild-type mice is in line with the notion that the triggering of ICOS by microenvironmental ICOSL plays a key role in the development of these cells. 

Collectively, these data suggest that OPN produced by TME favors tumor metastasis by interacting with stromal ICOSL; these activities are dominantly inhibited by ICOS. At the light of the important role played by both host OPN and ICOSL in metastasis development, further experiments using double OPN-KO and ICOSL-KO mice would be useful to sustain our findings.

The multiple interaction spots of OPN/ICOSL detected by PLA in human melanoma metastases, compared to those in primary tumors, support the hypothesis the binding between these two molecules plays a role in metastasis, but appears to contradict our in vitro finding that melanoma cells expressing low levels of ICOSL are prone to survive in attachment-independent conditions and display increased metastatic capacity. We can speculate that low expression of ICOSL in the primary tumor might favor detachment of tumor cells, their survival in the blood stream, and their extravasation at the metastatic site, since these cells would be less sensitive to the dominant negative inhibition triggered by ICOS. At the metastasis site, the ICOSL/OPN interaction might thus be crucial for engraftment of the metastasis, possibly taking advantage of a positive feedback loop, since previous findings show that boosting ICOSL expression in 4T1 cells increases their expression of OPN [17]. This opens an opposite scenario, not mutually exclusive with the previous one, in which OPN, which is one of the most abundantly expressed genes in melanoma metastases, acts as a chemoattractant for circulating ICOSL-positive tumor cells, and thus promotes development of the premetastatic niche by interacting with its multiple receptors [33,34]. Beside its effect on tumor cell migration and survival, the network also plays a role in tumor neoangiogenesis, since OPN induces—while ICOS inhibits—angiogenesis. 

Targeting this network may exert an antitumor effect by acting at multiple levels and addressing important unmet clinical needs, since approximatively 60% of patients do not achieve any significant therapeutic response, and a substantial proportion of responders relapse within 2 years [35]. 

ICOS-Fc treatment of mice carrying established primary melanomas decreases infiltrating Treg cells [36,37]. Anti-PD1 and anti-CTLA4 treatments are effective in melanoma immune therapy and, intriguingly, their effectiveness is marked by increased expression of ICOS [38]. Therefore, ICOS itself may play a role as an effector molecule recruited by this therapy, possibly by acting at several levels in the ICOS/ICOSL/OPN network. Recently, OPN has been shown to act as an immune checkpoint that negatively regulates T-cell activation and decreases the effectiveness of anti-PD-1-based immune therapy [39]. OPN neutralization may thus be another approach to increasing the efficacy of PD-1-based immune checkpoints blockade (ICB) immunotherapy in responding patients, and to overcome resistance to ICB immunotherapy in nonresponding patients [40]. 

Finely tuning/interfering with the OPN/ICOS/ICOSL network may represent a next-generation immunotherapy either for patients resistant to conventional therapies (i.e., ICB). 

This model may be relevant for other cancer types too. Accumulative evidence suggests that OPN is one of the most potent metastasis-associated proteins during the progression of other cancer types such as breast, colon, lung, pancreatic, renal, and esophageal cancer [33]. Cancer is often linked to an expansion of MDSCs/Tregs [41,42], in which OPN/ICOSL/ICOS trio may be involved. Given that the frequencies of MDSCs/Tregs have also been negatively correlated with the response to immunotherapy, targeting these cells in cancer patients may be a viable therapeutic approach to reverse immune escape and to maximize immune-based treatments. 

Lastly, many investigations have been performed to decipher the multiple signaling pathways and gene expression profiles induced by OPN by focusing on its binding to integrins and CD44. To the best of our knowledge, no studies have been performed yet investigating OPN/ICOSL intracellular signaling pathways. This research is necessary to improve the current understanding and to unveil the precise role of OPN/ICOSL interaction in tumor microenvironment remodeling, in tumor progression and metastasis development. These studies may be relevant to dissect pathogenetic roles and to identify new therapeutic targets. 

## Data Availability

The data shown in this article are available upon request from the corresponding author.

## Supplementary Materials

The following are available online at www.mdpi.com/article/10.3390/biomedicines10010051/s1: Figure S1: Gating strategy to define M-MDSC in lung; Figure S2: Gating strategies defining regulatory T cells (Treg) in lungs; Figure S3: Percentages of m-MDSC and Tregs in lungs of tumor-free bearing mice.
