# Supplementary material for: Inducible T-Cell Costimulator Ligand Plays a Dual Role in Melanoma Metastasis upon Binding to Osteopontin or Inducible T-Cell Costimulator
Source: Biomedicines. 2021 Dec 27;10(1):51. doi: 10.3390/biomedicines10010051 (PMC8772802; doi:10.3390/biomedicines10010051)
Supplement: Supplementary file 1 [file biomedicines-10-00051-s001.zip › biomedicines-1504392-supplementary.pdf]

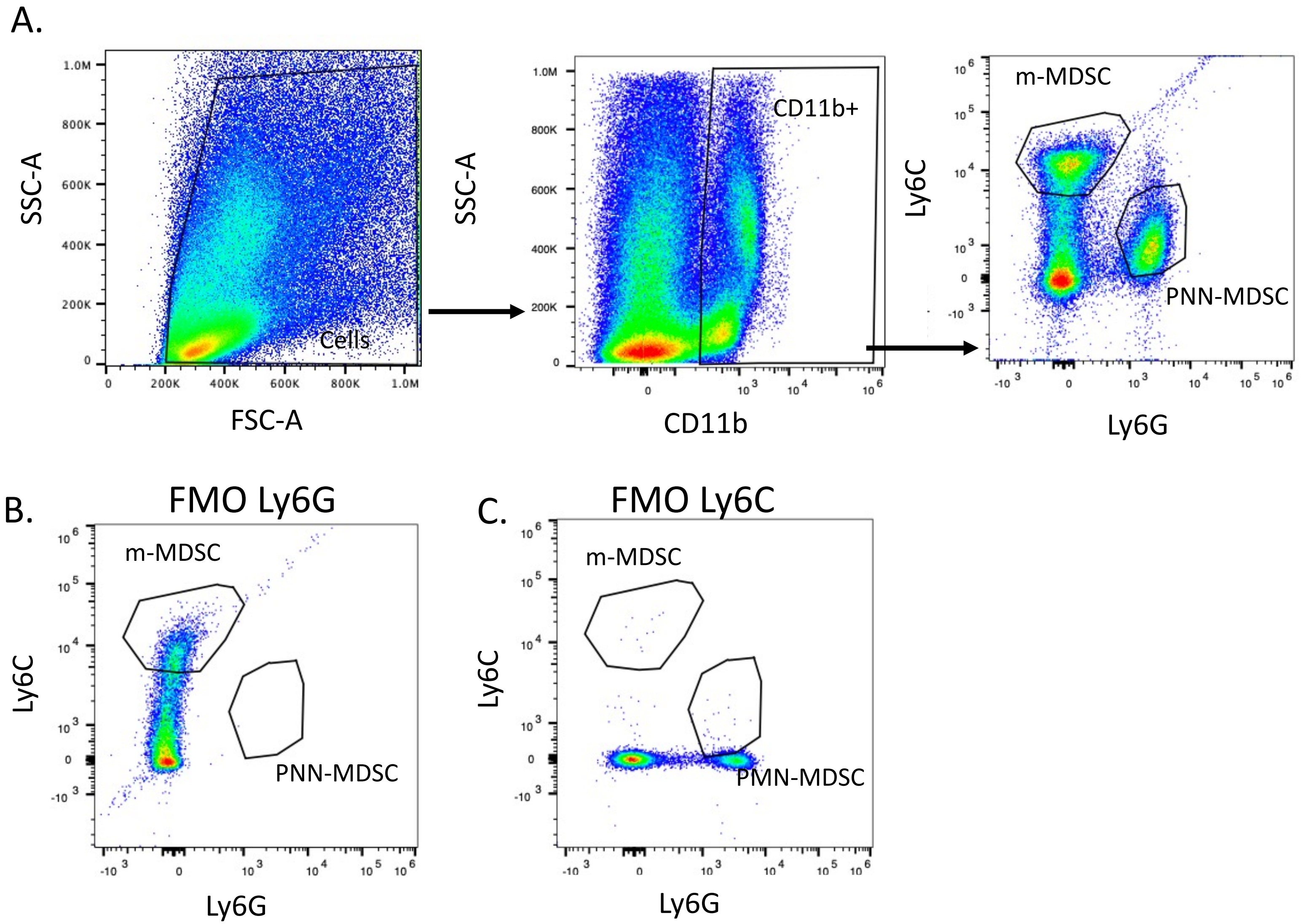

Supplementary Figure S1. Gating strategies defining M-MDSC in lungs. After the identification of the cells using the morphological parameters (FSC-A vs SSC-A), MDSC were identified as CD11b$^+$ cells and M-MDSC were defined as Ly6C-positive and Ly6G-negative cells. B) Fluorescence minus one (FMO) of Ly6G and C) FMO of Ly6C.

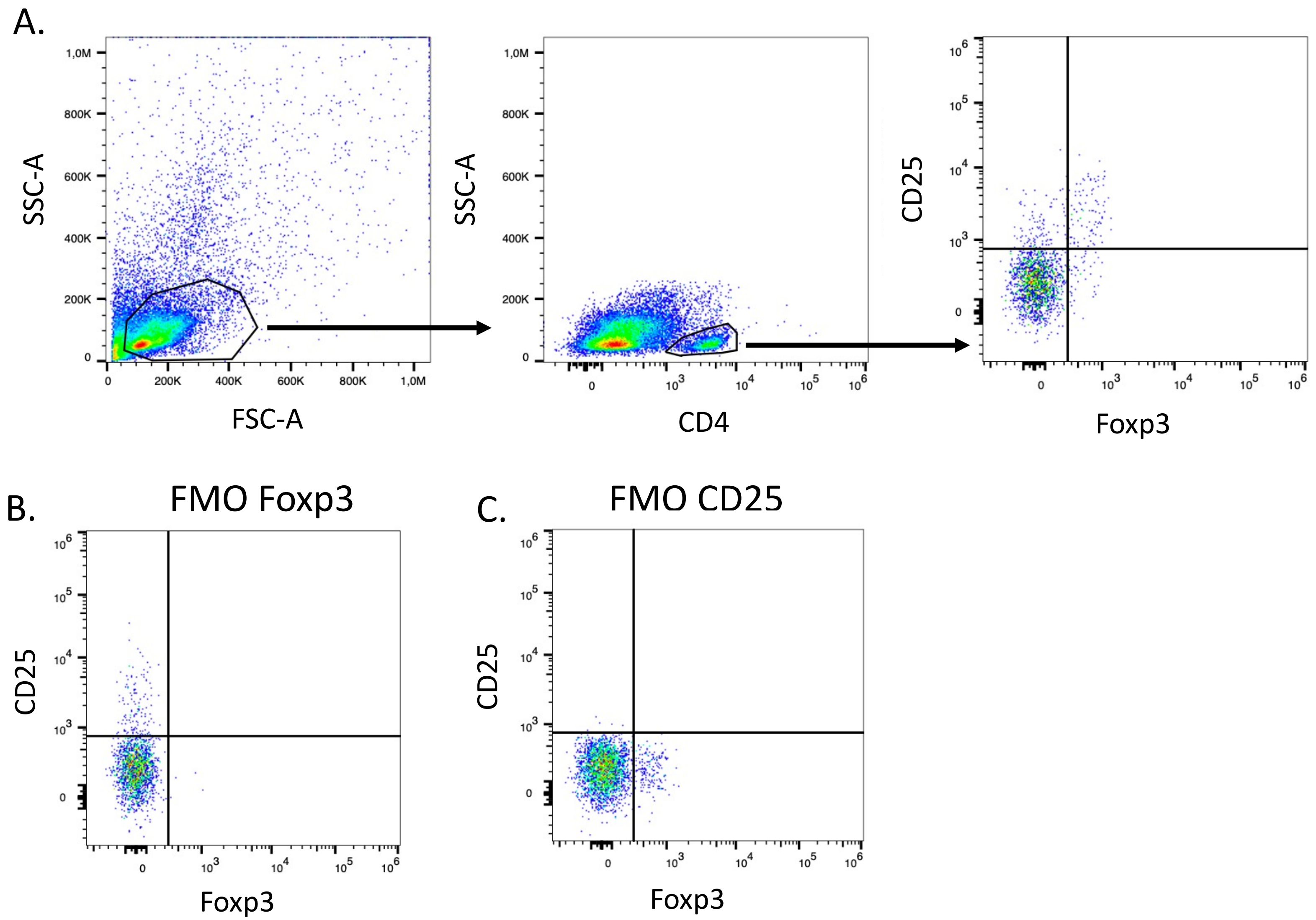

Supplementary Figure S2. A) Gating strategies defining regulatory T cells (Treg) in lungs. Cells were gated on CD4^{+} T cells after morphological discrimination (FSC-A vs. SSC-A), and Tregs are shown as CD25^{+}Foxp3^{+} cells, B) fluorescence minus one (FMO) of Foxp3 and C) FMO of CD25 markers.

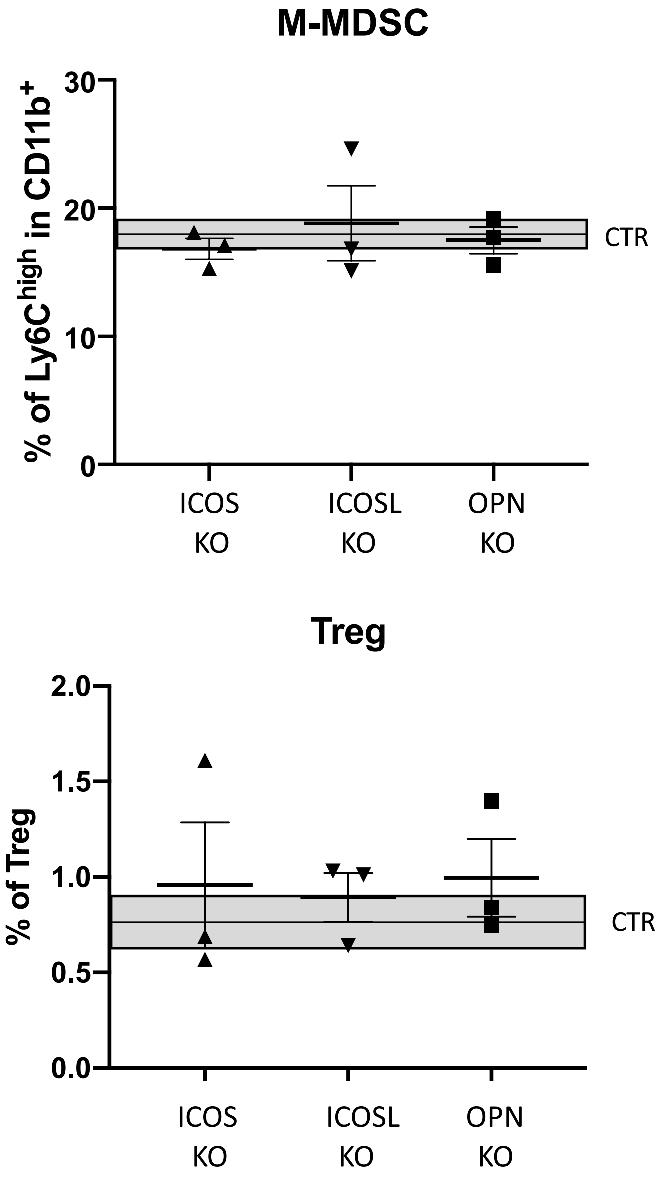

Supplementary Figure S3. Percentages of m-MDSC and Tregs in lungs of tumor free bearing mice.
